# Grand Challenges in Fungi-Plant Interactions

**DOI:** 10.3389/ffunb.2021.750003

**Published:** 2021-09-01

**Authors:** Raffaella Balestrini

**Affiliations:** National Research Council, Institute for Sustainable Plant Protection (CNR, IPSP), Turin, Italy

**Keywords:** mycorrhizal fungi, symbiosis, invasion, endophyte, abiotic stress

## Introduction

Fungi develop interactions with plants, ranging from detrimental to beneficial (mutualistic) associations, playing a major role in natural and agricultural ecosystems (Bennett and Cahill, 2016). These interactions may have in fact a great impact on the agriculture, the environment, and, consequently, the economy. Indeed, fungi have the potential to affect plant community structure, limiting the presence of some species and promoting others (Bennett and Cahill, 2016). Starting with plant pathogenic fungi, they are a relevant threat to crop production and food security and safety. Indeed, these effects are expected to worsen in the context of climate change scenario. However, it is worth note that rather than being additive, the presence of an abiotic stress can have the effect of reducing or enhancing susceptibility to a pathogen, and vice versa. Plants have evolved diverse defense mechanisms to cope with these detrimental interactions. Conversely, beneficial plant-associated fungi are broadly distributed and lead to benefits for the plant by supplying nutrients and increasing plant stress tolerance or disease resistance. Mycorrhizal fungi, i.e., a group of different fungal taxa, form symbiotic associations with roots of about 90% of all plant species, providing plants with mineral nutrients in exchange for fixed carbon (Balestrini and Lumini, 2018). Further research on the mechanisms involved in both pathogenic and mutualistic interactions, as well as the subtle differences that lead to the different result, is nowadays one of the most interesting topics in plant but also in fungal sciences. Particularly, novel information could be exploited to optimize beneficial associations to develop new plant protection strategies. Recent studies have also pointed the attention on the role of plant-associated microorganisms in the plant trade-off between growth and defense, opening new perspective in their use in sustainable agriculture (Bastías et al., 2021).

## Challenge in Unveiling the Role of Beneficial Soil Fungi to Cope With Multiple Environmental Stresses

Stress combinations instead of individual stresses have been recognized as realistic threats faced by plants (Suzuki et al., 2014; Pandey et al., 2015). Understanding the mechanisms involved in plant responses to multiple simultaneous stresses (biotic and abiotic) is crucial for the development of broad-spectrum strategies applicable for the improvement of tolerance and resilience in crops. Recent evidence shows that plants respond to combination of stresses by activating a program of gene expression, which differs from their single-stress responses and is related to the exact environmental conditions encountered (Atkinson and Urwin, 2012; Rejeb et al., 2014). In the context of the development of sustainable agricultural systems with a positive impact on the safety, security and quality of foodstuff, soil biologists have recently highlighted the potential of the interactions between roots and soil microorganisms to improve the plant's tolerance to abiotic (i.e., cold, drought, flooding, nutritional deficit) and biotic disturbances (i.e., pathogens and pests). In this context, the potential of soil fungi as natural fertilizers and pesticides is enormous. Despite these positive aspects, limited information is available on the role of microorganisms in a natural context where different biotic and abiotic stresses occur simultaneously. The outcome of these overlapping interactions is environment-dependent, and interactions may be beneficial or harmful depending on conditions, and the effects are often not sufficiently stable for practical application. The possibility to use diverse plant/microorganism combinations have already enabled the identification of microbial species that could be used to improve tolerance and resilience for a specific stress condition in several crops including tomato, showing species-specific effects (Chitarra et al., 2016; Volpe et al., 2018). A relevant research topic is to verify the microbial communities associates to several genotypes with contrasting behavior. It has been recently demonstrated that soil type as well as domestication and breeding can strongly affect root exudate composition with differences among the genotypes of the wheat domestication groups that were correlated with rhizosphere-metabolite composition (Iannucci et al., 2017), suggesting that this might have an impact on root-associated microorganisms.

## Challenge in Understanding Mechanisms of Plant-Fungus Interactions: Pot Experiments VS. Field

Simplified systems (i.e., pot experiments) are not adequate to capture all the variations present in nature and may not mirror interaction dynamics in natural ecosystems. Looking at plant-fungal interactions, the outcomes may depend on the environmental conditions, the identity of the partners, and/or the neighboring plants (Bennett and Cahill, 2016). Additionally, knowledge on the mechanisms by which soil fungi alter plant community structure, as well as the temporal dynamics on the interactions, is not still largely investigated and results are mainly derived from short-term pot experiments (Bennett and Cahill, 2016 and references therein). Considering individual plants, to quantify the plant response (i.e., considering plant traits correlated to performance as well as yield) to the colonization by selected beneficial microorganisms (individually and in combination) in multi-stress environments is an important challenge to optimize the exploitation of these microorganisms as well as to verify the impact of the considered inoculum on the microbial communities already present in the soil.

## Challenge to Unveiling the Diverse “Faces” of Fungal Endophytes

Endophytes (mainly bacteria and fungi) inhabit plant tissues for at least a part of their life cycle asymptomatically (Kaul et al., 2016; White et al., 2019). They have already been investigated as a novel source of fungal metabolites with diverse potential benefits in medical field and as microorganisms with a role in improving plant tolerance to environmental stresses (Kaul et al., 2016; Balestrini et al., 2021). Notwithstanding these positive aspects, some endophytes can be live with the host plants for several time in a latent phase, without showing disease symptoms (Marsberg et al., 2017). This relationship eventually changes when the host (i.e., the plant tissue) becomes favorable for disease development (Songy et al., 2019). Grapevine trunk diseases (GTDs) are one of the most relevant threats to vineyard sustainability. Interestingly, GTD-associated fungi can act as endophytes for several years until the appearance of the first symptoms, suggesting that abiotic conditions, mainly temperature and water stresses, may be involved in the development of the GTD symptoms. This is a relevant challenge considering that the frequency of these abiotic conditions is expected increase according to the climate change scenario (Songy et al., 2019).

Indeed, endophytes have long been considered as a potential source of biocontrol agents (White et al., 2019). Species generally considered to be aggressive plant pathogens such as *Botrytis* spp. were shown to be present as endophytes in many plant species without causing any disease (van Kan et al., 2014; Shaw et al., 2016). *Verticillium* genus comprises diverse species of which *V. dahliae* is the most relevant plant pathogen that infects hundreds of plants hosts including numerous crops. While half of the species are recognized as plant pathogens, other *Verticillium* spp. are known as saprophytes or endophytes (Malcolm et al., 2013; Shi-Kunne et al., 2018). Information about the mechanisms involved in these plant-microbe interactions is lacking and the potential of these endophytic “plant pathogens” as biocontrol agents is still unexplored.

## Challenge in Investigating the Interactions Between Invasive VS. Native Organisms

Plants in their native niches are involved in a network of interactions with fungi, both pathogens and mutualists. The process of human transport and invasion of plants and fungi alters these interactions through the loss of the native interactions and the establishment of novel ones between natives and aliens and between aliens from different origins. Although both plants and fungi are important groups of invasive species, their interactions are relevant actors of the impact of their invasions at ecological and evolutionary level (Mitchell et al., 2006; Pringle et al., 2009). Invasion processes provide an increase in complexity at ecological, and biological level, leading to the creation of novel associations as well as to the disruption of native symbiont interactions. An important point that should be addressed is to highlight the impact of invasive pathogens on mutualistic interactions (Zampieri et al., 2017; Gonthier et al., 2019). Additionally, the identification of the native habitat of fungi is still a challenge and most invasive fungi remain still undetected (Dickie et al., 2017). For this reason, wide surveys on biodiversity are essential to determine species origins and highlight invasions.

## Conclusions

Several advancements have been achieved and an important body of knowledge has been generated on different biological, physiological, and ecological aspects of the interactions between plants and beneficial microorganisms, suggesting a relevant role to improve plant tolerance and resilience. However, the functional potential of plant associated microbiota remains largely unknown and several aspects of these interactions, including the mechanisms at the basis of the regulation of plant defenses in the presence of beneficial microorganisms, have not been clearly elucidated. The use of beneficial plant-associated microorganisms to improve plant resistance might be also useful for those crop cultivars where genetic resistance has been overcome by pathogens.

## Author Contributions

RB conceived and wrote the manuscript and approved it for publication.

## Conflict of Interest

The author declares that the research was conducted in the absence of any commercial or financial relationships that could be construed as a potential conflict of interest.

## Publisher's Note

All claims expressed in this article are solely those of the authors and do not necessarily represent those of their affiliated organizations, or those of the publisher, the editors and the reviewers. Any product that may be evaluated in this article, or claim that may be made by its manufacturer, is not guaranteed or endorsed by the publisher.

## References

[B1] Atkinson N. J. Urwin P. E. (2012). The interaction of plant biotic and abiotic stresses: from genes to the field. J. Exp. Bot. 63, 3523–3543. 10.1093/jxb/ers100 22467407

[B2] Balestrini R. Brunetti C. Cammareri M. Caretto S. Cavallaro V. Cominelli E.. (2021). Strategies to modulate specialized metabolism in Mediterranean crops: From molecular aspects to field. Int. J. Mol. Sci. 22:2887. 10.3390/ijms22062887 33809189 PMC7999214

[B3] Balestrini R. Lumini E. (2018). Focus on mycorrhizal symbioses. Appl. Soil Ecol. 123, 299–304. 10.1016/j.apsoil.2017.09.001

[B4] Bastías D. A. Gianoli E. Gundel P. E. (2021). Fungal endophytes can eliminate the plant growth–defence trade-off. New Phytol. 230, 2105–2113. 10.1111/nph.17335 33690884

[B5] Bennett J. A. Cahill J. F. (2016). Fungal effects on plant-plant interactions contribute to grassland plant abundances: evidence from the field. J. Ecol. 104, 755–764. 10.1111/1365-2745.12558

[B6] Chitarra W. Pagliarani C. Maserti B. Lumini E. Siciliano I. Cascone P.. (2016). Insights on the impact of arbuscular mycorrhizal symbiosis on tomato tolerance to water stress. Plant Physiol. 171, 1009–1023. 10.1104/pp.16.00307 27208301 PMC4902612

[B7] Dickie I. A. Bufford J. L. Cobb R. C. Desprez-Loustau M.-L. Grelet G. Hulme P. E.. (2017). The emerging science of linked plant–fungal invasions. New Phytol. 215, 1314–1332. 10.1111/nph.14657 28649741

[B8] Gonthier P. Giordano L. Zampieri E. Lione G. Vizzini A. Colpaert J. V.. (2019). An ectomycorrhizal symbiosis differently affects host susceptibility to two congeneric fungal pathogens. Fungal Ecol. 39, 250–256. 10.1016/j.funeco.2018.12.008

[B9] Iannucci A. Fragasso M. Beleggia R. Nigro F. Papa R. (2017). Evolution of the crop rhizosphere: impact of domestication on root exudates in tetraploid wheat (*Triticum turgidum* L.). *Front. Plant Sci*. 8:2124. 10.3389/fpls.2017.02124 29326736 PMC5733359

[B10] Kaul S. Sharma T. Dhar M. K. (2016). Omics Tools for better understanding the plant-endophyte interactions. *Front*. Plant Sci. 7:955. 10.3389/fpls.2016.00955 27446181 PMC4925718

[B11] Malcolm G. M. Kuldau G. A. Gugino B. K. del Mar Jiménez-Gasco M. (2013). Hidden host plant associations of soilborne fungal pathogens: an ecological perspective. Phytopathology 103, 538–544. 10.1094/PHYTO-08-12-0192-LE 23301815

[B12] Marsberg A. Kemler M. Jami F. Nagel J. H. Postma-Smidt A. Naidoo S.. (2017). *Botryosphaeria dothidea*: a latent pathogen of global importance to woody plant health. Mol. Plant Pathol. 18, 477–488. 10.1111/mpp.12495 27682468 PMC6638292

[B13] Mitchell C. E. Agrawal A. A. Bever J. D. Gilbert G. S. Hufbauer R. A. Klironomos J. N.. (2006). Biotic interactions and plant invasions. *Ecol*. Lett. 9, 726–740. 10.1111/j.1461-0248.2006.00908.x 16706916

[B14] Pandey P. Ramegowda V. Senthil-Kumar M. (2015). Shared and unique responses of plants to multiple individual stresses and stress combinations: physiological and molecular mechanisms. Front. Plant Sci. 6:723. 10.3389/fpls.2015.00723 26442037 PMC4584981

[B15] Pringle A. Bever J. D. Gardes M. Parrent J. L. Rillig M. C. Klironomos J. N. (2009). Mycorrhizal symbioses and plant invasions. Ann. Rev. Ecol. Evol. Syst. 40, 699–715. 10.1146/annurev.ecolsys.39.110707.173454

[B16] Rejeb I. B. Pastor V. Mauch-Mani B. (2014). Plant responses to simultaneous biotic and abiotic stress: molecular mechanisms. Plants 3, 458–475. 10.3390/plants3040458 27135514 PMC4844285

[B17] Shaw M. W. Emmanuel C. J. Emilda D. Terhem R. B. Shafia A. Tsamaidi D.. (2016). Analysis of cryptic, systemic *Botrytis* infections in symptomless hosts. Front. Plant Sci. 7:625. 10.3389/fpls.2016.00625 27242829 PMC4861902

[B18] Shi-Kunne X. Faino L. van den Berg G. C. M. Thomma B. P. H. J. Seidl M. F. (2018). Evolution within the fungal genus *Verticillium* is characterized by chromosomal rearrangement and gene loss. Environ. Microbiol. 20, 1362–1373. 10.1111/1462-2920.14037 29282842

[B19] Songy A. Fernandez O. Clément C. Larignon P. Fontaine F. (2019). Grapevine trunk diseases under thermal and water stresses. Planta 249, 1655–1679. 10.1007/s00425-019-03111-8 30805725

[B20] Suzuki N. Rivero R. M. Shulaev V. Blumwald E. Mittler R. (2014). Abiotic and biotic stress combinations. New Phytol. 203, 32–43. 10.1111/nph.12797 24720847

[B21] van Kan J. A. L. Shaw M. W. Grant-Downton R. T. (2014). Botrytis: thugs or endophytes? Mol. Plant Pathol. 15, 957–961. 10.1111/mpp.12148 24754470 PMC6638755

[B22] Volpe V. Chitarra W. Cascone P. Volpe M. G. Bartolini P. Moneti G.. (2018). The Association with two different arbuscular mycorrhizal fungi differently affects water stress tolerance in tomato. *Front*. Plant Sci. 9:1480. 10.3389/fpls.2018.01480 30356724 PMC6189365

[B23] White J. F. Kingsley K. L. Zhang Q. Verma R. Obi N. Dvinskikh S. (2019), Review: endophytic microbes their potential applications in crop management. Pest. Manag. Sci. 75, 2558–2565. 10.1002/ps.5527. 31228333 PMC6771842

[B24] Zampieri E. Giordano L. Lione G. Vizzini A. Sillo F. Balestrini R.. (2017). A nonnative and a native fungal plant pathogen similarly stimulate ectomycorrhizal development but are perceived differently by a fungal symbiont. New Phytol. 213, 1836–1849. 10.1111/nph.14314 27870066

